# Cancer Trajectories at the End of Life: is there an effect of age and gender?

**DOI:** 10.1186/1471-2407-8-127

**Published:** 2008-05-02

**Authors:** Massimo Costantini, Monica Beccaro, Irene J Higginson

**Affiliations:** 1 Regional Palliative Care Network, National Cancer Research Institute, Largo R. Benzi, 10; 16132 Genova, Italy; 2 Department of Palliative Care, Policy and Rehabilitation, King's College London, School of Medicine, Weston Education Centre, Cutcombe Rd, London SE5 9RJ, UK

## Abstract

**Background:**

Few empirical data show the pattern of functional decline at the end of life for cancer patients, especially among older patients.

**Methods:**

In a mortality follow-back survey (the Italian Survey of the Dying of Cancer – ISDOC) a random sample of 1,271 lay caregivers were interviewed, at a mean of 234 days after bereavement. The main outcome was number of days before death when the patient experienced a permanent functional decline.

**Results:**

1,249 (98%) caregivers answered the question about patient's function. The probability to be free from a functional disability was high (94%) 52 weeks before death, but was lower for older age groups (15% for those aged 85 or more) and women (8%). It remained stable until 18 weeks before death, then fell to 63% at 12 weeks and 49% at 6 weeks before death (among those aged 85 or more the figures were 50% and 41%). The pattern was consistent across sub-groups, except for patients affected by Central Nervous System tumors who experienced a longer, slower functional decline.

**Conclusion:**

This study provides empirical support for the declining trajectory in cancer, and suggests that the decline commences at around 12 weeks in all age groups, even among patients over 85 years.

## Background

Although functional status is a well know prognostic indicator in cancer [1-3], the pattern of decline is less well understood. In 2003, Lunney et al. proposed a model of theoretical patterns of functional decline at the end of life, showing four main trajectories, sudden death, terminal illness, organ failure and frailty [4]. The authors analysis, and a previous study [5], suggested most people dying from cancer fell into the category of 'terminal illness', which showed high function early in their final year, but marked more disability three months prior to death. These results, derived from cross-sectional studies, have not yet been re-tested in other research with different designs. The trajectories are now being used to underpin government strategies in many countries to help determine eligibility for hospice and palliative care services. Following USA trends, European Clinicians are being encouraged to use changing function and time (such as in the UK End of Life Strategy) to decide when to refer patients to hospice and palliative care services. However, because of demographic changes and improvements in treatment, cancer patients increasingly are older [6,7]. Most of the studies examining the role of functional status in prognosis have excluded older people, with the assumption that disability due to co-morbidity will lead to misclassification [3]. Therefore, in this study we aimed to provide empirical data to test the hypothesis of a trajectory of stable disease followed by rapid decline in cancer at the end of life. We wished to test whether the trajectories were universal, or were they different among patients with different ages, gender and primary cancer diagnosis.

## Methods

A 2-stage probability sample was used to estimate end-of-life outcomes of approximately 160,000 annual cancer deaths in Italy. In the 1^st ^sampling stage, 30 out of 197 existing Local Health Districts (LHD) were randomly selected. In the 2^nd ^stage, a fixed proportion of cancer deaths aged 18 years or more were randomly drawn from each LHD, providing a sample of 2,000 death certificates for people who died from cancer between March 2002 and June 2003. For each case included in the survey, the non-professional caregiver, who was defined as the closest and the best informed person about the patient's last three months of life, was identified. A professional caregiver was identified for 57 deceased (3%) who were alone and without any non-professional support. A letter was sent to all identified caregivers to inform them of the study aims and obtain formal consent to be interviewed. A trained interviewer contacted the identified caregiver to discuss the interview in detail. Information regarding the methodology of the survey has been published in a previous article [8].

The interviewer met the caregiver, usually in his/her home, to conduct a semi-structured interview using an adapted version of the VOICES questionnaire [9]. A specific section of the interview dealt with the major functional decline of the patient. More specifically, the caregiver was asked:

• When did the patient start to need assistance or help to wash, dress, eat or walk around the house?" (Days before death).

During specific training settings, the interviewers were instructed to specify the caregiver that the question referred to any cause of disability, not only cancer.

The study design was approved by the Ethical Committee of National Cancer Institute of Genoa, and, according to the Italian law on use and processing of health data, a notification of the study design and procedures was sent to the Italian Data Protection Commission.

### Statistics

The probability to be free from a major and permanent functional disability has been modelled in the 52 weeks before death using life-table statistical analysis. The last year of life was subdivided in 26 intervals of two weeks. The cumulative probability to be free from functional disability was estimated at the end of each interval and for each point the standard error calculated.

All analyses were performed using SUDAAN version 9.0.1 (Research triangle Institute, Research Triangle Park, NC). This software, for the point and standard error statistic estimation, takes into account the unequal probability selection of observations, stratification and clustering of observations. Sampling weights were introduced to obtain unbiased weighted point estimates and standard error of the target population. Chi-square test for heterogeneity was used to examine the distribution of caregiver relationship by the characteristics of the study sample.

## Results

An interview was conducted with 1,289 (67.8%) of the 1,900 identified caregivers at an average of 234 days after the patients' death (range 103–374). Six patients whose cause of death was not cancer and twelve without a terminal phase of disease were excluded from all the statistical analysis, leaving a total sample of 1,271 valid interviews, and of these 1,249 (98.3%) caregivers answered the question about patient's function.

Responding caregivers were a child (46.4%), the spouse (30.3%), another family member or a friend (20.4%), or a health professional for patients with no lay caregiver (2.9%). Most cancer patients were at least 65 years old at death (80.8%) and 57.4% were men. About two-third (67%) completed only primary schools. Tumors from the digestive, respiratory or genitourinary system accounted for 72% of all cancer deaths. (Table 1). There was a significant relationship between responding caregiver and age, gender, level of patient education and primary tumor (Table 1). Spouse respondents more commonly reported about patients who were younger, men, with the highest education level and suffering from respiratory primary tumors. Conversely, children more frequently reported about patients who were older, women, with the lowest educational level. Similarly, for patients without caregivers, health professionals more frequently reported about those who were older, women, with the lowest educational level.

**Table 1:** Characteristics of the study sample by caregiver relationship

	TOTAL		Spouse		Daughter – Son		Other Relatives		Health Professionals		
			
	N.	%	N.	%	N.	%	N.	%	N.	%	
AGE AT DEATH (years)											
18–64	242	19.2	128	32.5	56	10.2	56	20.9	2	4.6	
65–84	785	64.1	237	64.1	391	68.6	139	54.9	18	51.2	
85 +	222	16.7	14	3.3	132	21.1	60	24.2	16	44.2	P < 0.001
GENDER											
Males	700	57.4	297	79.9	281	49.3	109	43.7	13	34.1	
Females	549	42.6	82	20.1	298	50.7	146	56.3	23	65.9	P < 0.001
EDUCATION (years)											
≤ 5	819	66.9	203	54.2	427	75.2	163	66.7	26	72.1	
6–8	224	17.1	86	21.8	86	14.0	45	16.6	7	19.8	
≥ 9	205	16.0	90	24.0	65	10.8	47	16.7	3	8.1	P < 0.001
PRIMARY TUMOR											
Digestive	453	35.6	120	30.4	237	39.7	87	35.2	9	25.9	
Respiratory	262	21.7	113	29.3	99	18.4	44	18.4	6	16.3	
Breast	124	10.0	29	7.8	52	9.1	30	11.7	13	40.3	
Genitourinary	184	14.8	58	16.3	85	14.5	38	13.9	3	6.8	
Haematological	91	7.5	26	7.0	45	8.2	17	6.7	3	6.8	
Nervous Central System	32	2.7	9	2.8	15	2.8	8	2.7	-	-	
Others	103	7.8	24	6.5	46	7.4	31	11.4	2	3.9	P = 0.003
TOTAL	1,249	100	379	100	579	100	255	100	36	100	

NOTE: all percentages are weighted column percentages

**Figure 1:**
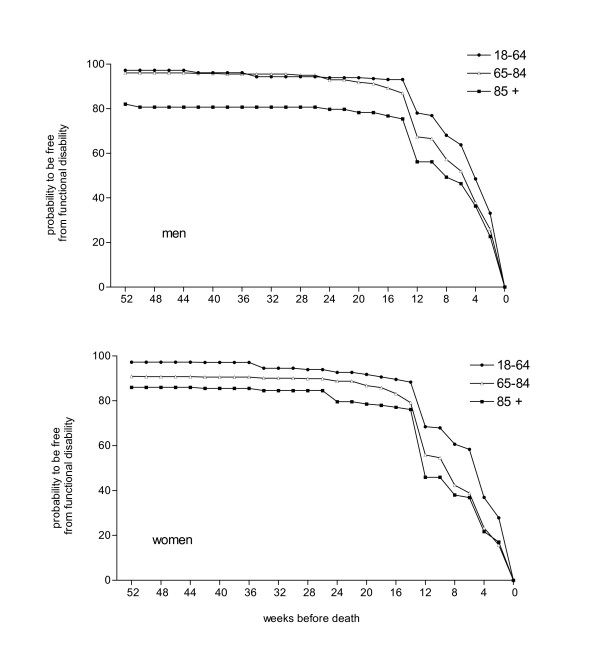
Probability to be free from functional disability in the year before death of cancer patients in men and women by age group.

For all cancer patients, the probability to be free from a major and permanent functional disability was very high (94%) 52 weeks before death. It decreased slightly during the next 34 weeks (Figure 1 and Table 2). Most patients became functionally disabled during the last 18 weeks of life. Their probability to be free from disability decreased from 88% (18 weeks before death) to 63% (12 weeks before death), to 49% (6 weeks before death), to 23% (2 weeks before death).

**Table 2:** Probability to be free from functional disability in the year before death of cancer patients by selected characteristics

		Probability to be free from functional disability (weeks before death)						
		
	N.	52 weeks % (95% CI)	24 weeks % (95% CI)	18 weeks % (95% CI)	12 weeks % (95% CI)	6 weeks % (95% CI)	4 weeks % (95% CI)	2 weeks % (95% CI)
Gender								
Men	700	95 (93–97)	92 (89–94)	91 (88–93)	68 (63–73)	53 (49–58)	39 (35–44)	27 (23–31)
Women	549	92 (89–94)	88 (84–91)	86 (82–89)	56 (51–62)	43 (37–48)	26 (21–31)	18 (15–22)
Age at death								
18–64	242	99 (96–99)	95 (91–97)	93 (89–96)	74 (68–80)	62 (55–68)	43 (36–50)	30 (25–36)
65–84	785	94 (92–96)	92 (89–94)	90 (86–92)	63 (58–68)	47 (43–51)	32 (28–36)	22 (19–25)
85 +	222	85 (79–90)	80 (74–85)	79 (73–84)	50 (42–58)	41 (34–47)	28 (23–33)	19 (14–24)
Primary Tumour								
Digestive	453	94 (92–96)	91 (88–94)	90 (87–92)	65 (60–70)	53 (48–58)	35 (30–40)	23 (19–27)
Respiratory	262	97 (93–99)	94 (90–96)	91 (85–95)	69 (61–76)	52 (45–59)	39 (32–46)	28 (23–34)
Breast	124	91 (83–95)	88 (80–93)	87 (80–91)	58 (49–67)	45 (35–55)	24 (15–37)	19 (12–29)
Genitourinary	184	93 (87–97)	87 (80–92)	85 (80–90)	59 (49–68)	41 (33–49)	28 (21–36)	15 (11–23)
Hematological	91	91 (83–95)	90 (82–94)	88 (80–93)	62 (52–72)	52 (42–62)	40 (32–49)	30 (22–40)
Nervous Central System	32	87 (67–96)	76 (59–87)	72 (56–84)	46 (26–68)	35 (19–55)	14 (5–31)	6 (2–19)
Others	103	92 (84–96)	90 (82–95)	90 (81–95)	60 (44–74)	42 (31–55)	37 (27–49)	28 (18–40)
All patients	1,249	94 (92–95)	90 (88–93)	88 (86–91)	63 (58–68)	49 (45–53)	33 (30–37)	23 (20–26)

When we examined the probability to be free from functional disability in the year before death, by gender, age and primary tumors (Table 2), two phenomena emerged. First, the patterns in all subgroups were similar, showing a period of stable, higher function followed by rapid decline starting at 12–18 weeks before death. The only remarkable exception is the group of patients affected by Central Nervous System (CNS) tumors who experienced a longer, slower functional decline starting 24 weeks before death. Second, a higher proportion of disabled cancer patients 52 weeks before death was observed for older age groups (15% for those aged 85 or more) and for women (8%). These differences between sub-groups remained throughout the trajectory until the last 2 weeks before death.

Figure 1 shows the probability to be free from functional disability in the 52 weeks before death in men and women by age group. The patterns of functional decline were comparable for all six subgroups and older age groups show a higher proportion of disabled cancer patients one year before death both among males and females.

## Discussion

These data, derived from a nationally representative mortality follow-back survey, provide an empirical confirmation of the validity of these trajectories for patients with incurable cancers. They confirm the general pattern of the trajectory and the timing of the onset of functional deterioration, about 3 – 4 months before death. Overall, our trajectory is very similar to that reported by Teno et al [5] for cancer patients. Both begin to decline at 6 months before death. However, the decline in Teno's et al. analysis is more rapid in the following 2–3 months, whereas our trajectory suggest that the probability of maintaining function is high during this period. Our data show a more rapid decline in the final 3 months of life, which is at greater variance from the trajectories of cerebrovascular disease, respiratory failure, congestive heart failure and diabetes, as suggested by Teno et al [5].

Limitations of this study include the nature of mortality follow-back surveys, being based on the retrospective views of lay caregivers, which are subject to recall bias, as well as representing the caregiver rather than the patient assessment. However, a review of the validity of this approach found that for objective, rather than subjective symptom and psychological, assessments caregiver reports can be a valid and reliable proxy for patient views [10]. Although the response rate was high (68%) for this kind of design, the results may be biased by the effects of the survey design. As reported in the multivariate analyses of the baseline data [8] a slightly lower response rate was observed for patients who died in hospital or nursing homes compared to home, and when the caregiver was the spouse. All these patients may have experienced higher levels of disability, and our results could have underestimated the total amount of disability. Another potential source of bias could derive from the different respondents, as the there were more children and health professionals respondents for the oldest patients. It may be that these respondents reported disability in a different way to spouses. However it is unlikely that this has affected the overall trajectories.

The main problem with the assessment in this study is that it considers the major loss of functional ability as a permanent outcome. Function may fluctuate among cancer patients at the end of life. Our interview did not assess this, but it is questionable whether any retrospective assessment could do so. Longitudinal prospective studies would be needed to assess more subtle changes, and to assess the trajectories of problems such as symptoms and psychological concerns, all of which might be important in determining need for palliative care.

## Conclusion

Despite its limitations, this study suggests that deteriorating function is an important prognostic indicator for all age groups. Co-morbidities probably explain the lower proportion of older patients with disability one year before death, but the pattern and starting point (at 12 weeks before death) is consistent across age-groups. Even among cancer patients over 85 years, a permanent reduction in function has a high probability to be associated with commencing the terminal phase of life. This simple indicator could be used by clinicians to revisit the potential benefits of aggressive treatments in cancer, initiate discussion with the patient and caregivers about end of life care preferences and referral to palliative care services. Caregivers and patients could also be given the information that functional decline indicates worsening prognosis when asking for information about prognosis. Such an indicator would be useful not only in hospitals, but also in the community and in nursing and residential homes, where increasingly older cancer patients are cared for.

## Competing interests

The three authors have no conflicts of interest, including specific financial interests and relationships and affiliation relevant to the subject matter or materials discussed in the manuscript.

## Authors' contributions

Study concept and design: MC, MB, IJH; acquisition of data: MC, MB; analysis and interpretation of data: MC, IJH; drafting of the manuscript: MC, IJH; critical revision of the manuscript for important intellectual content: MC, MB, IJH; statistical analysis: MC, IJH; obtaining funding: MC, MB; administrative, technical, or material support: MB. All authors read and approved the final manuscript.

## Pre-publication history

The pre-publication history for this paper can be accessed here:


